# Supplementary material for: In Vitro Digestion and Fermentation of Different Ethanol-Fractional Polysaccharides from Dendrobium officinale: Molecular Decomposition and Regulation on Gut Microbiota
Source: Foods. 2024 May 27;13(11):1675. doi: 10.3390/foods13111675 (PMC11172086; doi:10.3390/foods13111675)
Supplement: Supplementary file 1 [file foods-13-01675-s001.zip › foods-2972459-supplementary.pdf]

In vitro digestion and fermentation of different ethanol-fractional polysaccharides from *Dendrobium officinale*: Molecular decomposition and regulation on gut microbiota

Lei Xu[a,b], Hua Zhu[a,b,d], Peng Chen[a,b], Zhenhao Li[c], Kai Yang[a,b], Peilong Sun[a,b], Fangting Gu[d], Jianyong Wu[d], Ming Cai[a,b,*]

[a]*Department of Food Science and Technology, Zhejiang University of Technology, Hangzhou, Zhejiang 310014, People's Republic of China*

[b]*Key Laboratory of Food Macromolecular Resources Processing Technology Research (Zhejiang University of Technology), China National Light Industry, People's Republic of China*

[c]*Longevity Valley Botanical Co., Ltd., Zhejiang 321200, People's Republic of China*

[d]*Department of Food Science & Nutrition, The Hong Kong Polytechnic University, Hung Hom, Kowloon, Hong Kong Special Administrative Region of the People's Republic of China*

Corresponding authors:

Dr. Cai Ming, Tel: (86) 571-88813778, E-mail: caiming@zjut.edu.cn

Supplementary pictures

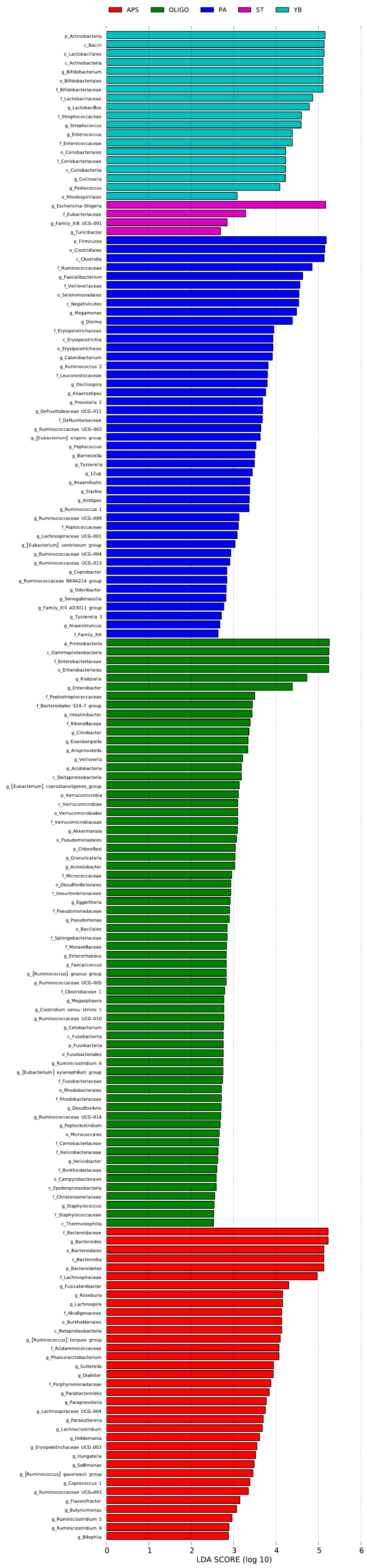

Fig. S1. Lefse analyses of gut microbiota after filtering of different samples

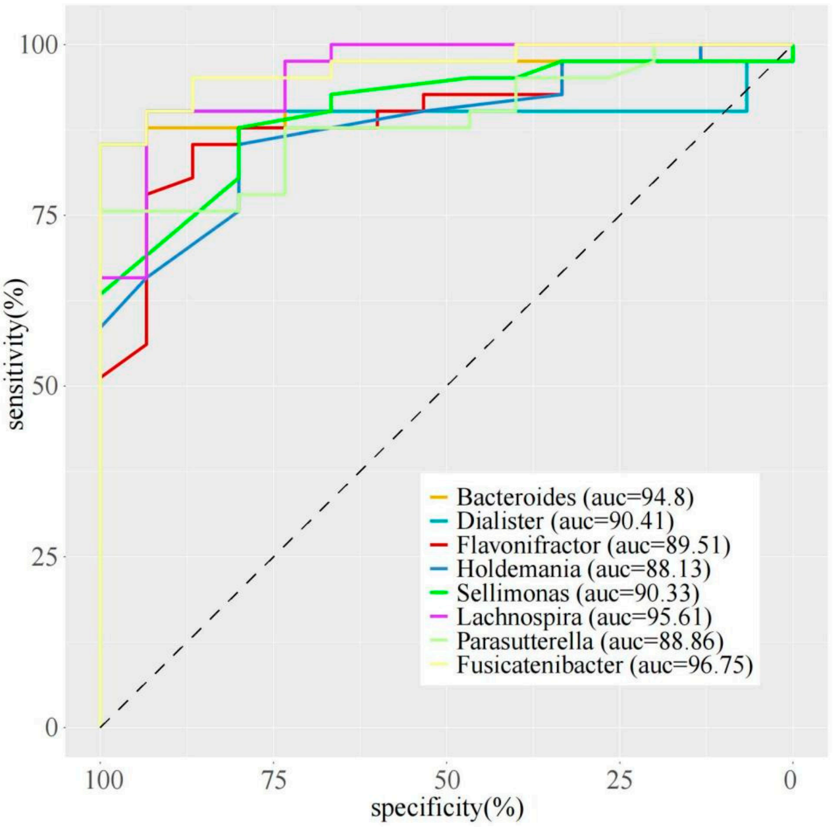

Fig. S2. ROC curves of 8 genus gut microbiota distinguishing between APS and non-APS groups

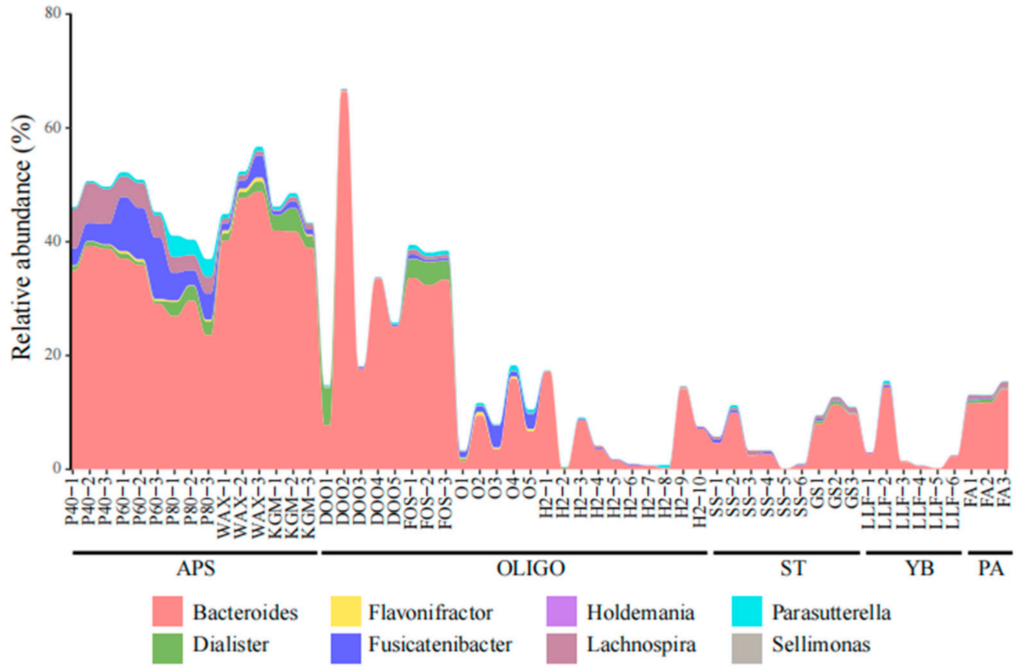

Fig. S3. The abundance of the eight selected gut microbiota in the different samples.

Supplementary tables

Table S1. Saliva, gastric and intestine simulated digestive fluid addition table.

Name	Chemical reagent	Dosage	Source
**Saliva electrolyte solution**	KCl	0.4 g	Aladdin, Shanghai, China
	NaCl	1.4 g	Aladdin, Shanghai, China
	Deion-water	200 mL	Lab made
**Simulated saliva fluid**	Saliva electrolyte solution	50 mL	Lab made
	Fresh centrifugal saliva	50 mL	Lab made
**Gastric electrolyte solution**	KCl	0.22 g	Aladdin, Shanghai, China
	NaCl	0.62 g	Aladdin, Shanghai, China
	$NaHCO_3$	0.12 g	Aladdin, Shanghai, China
	$CaCl_2$	0.05 g	Aladdin, Shanghai, China
	Deion-water	200 mL	Lab made
	1 mol/L HCl	to pH 2.0	Aladdin, Shanghai, China
**Simulated gastric fluid**	Porcine pepsin	35.4 mg	Yuanye, Shanghai, China
	Gastric lipase	37.5 mg	Yuanye, Shanghai, China
	1 mol/L CH_3COONa	1 mL	Aladdin, Shanghai, China
	Gastric electrolyte solution	150 mL	Lab made
	1mol/L HCl	to pH 3.0	Aladdin, Shanghai, China
**Intestinal electrolyte solution**	KCl	0.065 g	Aladdin, Shanghai, China
	NaCl	0.54 g	Aladdin, Shanghai, China
	$CaCl_2$	0.033 g	Aladdin, Shanghai, China
	Deion-water	100 mL	Lab made
	1 mol/L NaOH	to pH 7.5	Aladdin, Shanghai, China
**Simulated intestinal fluid**	Cholate (4%, w/w)	200 g	Yuanye, Shanghai, China
	Trypsin	6.5 g	Yuanye, Shanghai, China
	Pancreatin	50 g	Yuanye, Shanghai, China
	Intestinal electrolyte solution	50 mL	Lab made
	1mol/L NaOH	to pH 7.5	Aladdin, Shanghai, China

Table S2. Large intestinal simulated fermentation fluid addition table

Name	Chemical reagent	Dosage	Source
**Vitamin I solution**	Vitamin B12	2 mg	Sigma Aldrich, Shanghai, China
	Vitamin H	2 mg	Sigma Aldrich, Shanghai, China
	Vitamin B9	10 mg	Sigma Aldrich, Shanghai, China
	Vitamin B6	30 mg	Sigma Aldrich, Shanghai, China
	Sulfanilic acid	6 mg	Aladdin, Shanghai, China
	Deion-water	40 mL	Lab made
**Metaphosphoric acid solution**	Metaphosphoric acid	2.5 g	Aladdin, Shanghai, China
	Deion-water	100 mL	Lab made
**Batanic acid solution**	Batanic acid	0.6464 g	Aladdin, Shanghai, China
	Metaphosphoric acid solution	100 mL	Lab made
**Simulated Large intestinal fluid**	KH_2PO_4	0.45 g	Aladdin, Shanghai, China
	K_2HPO4	0.45 g	Aladdin, Shanghai, China
	NaCl	0.05 g	Aladdin, Shanghai, China
	$CaCl_2 \cdot 2H2O$	0.064 g	Aladdin, Shanghai, China
	$MgSO_4 \cdot 7H2O$	0.09 g	Sigma Aldrich, Shanghai, China
	Yeast extract	2.5 g	Sigma Aldrich, Shanghai, China
	Tryptone	10 g	Sigma Aldrich, Shanghai, China
	Haemachrome	2 mL	Sigma Aldrich, Shanghai, China
	Batanic acid solution	100 mL	Sigma Aldrich, Shanghai, China
	L-cysteine	1 g	Yuanye, Shanghai, China
	Vitamin I solution	0.2 mL	Sigma Aldrich, Shanghai, China
	Deion-water	1000 mL	Lab made

Table S3. Statistical analysis of partial gut microbiota.

	Group					
	**BLANK-0**	**BLANK**	**P40**	**P60**	**P80**	**FOS**
The ratio of *Bacteroides* to *Firmicutes* (B/F)	0.8751	0.804 ± 0.088	1.022 ± 0.123	1.228 ± 0.144 *	0.878 ± 0.036	0.890 ± 0.018
Abundance of *Eschrichia-Shigella*	0.0962	0.0825 ± 0.014	0.0658 ± 0.014	0.0478 ± 0.025 *	0.0621 ± 0.004	0.1002 ± 0.022
Proteobact	0.0279	0.0434 ± 0.0019 *	0.0179 ± 0.0005	0.0549 ± 0.0037 *	0.0257 ± 0.0044	0.0175 ± 0.0027
Megamouas	0.0613	0.0455 ± 0.002	0.0395 ± 0.002	0.0048 ± 0.001 *	0.0078 ± 0.002 *	0.0581 ± 0.019
Prevotella 9	0.0802	0.0825 ± 0.015	0.0758 ± 0.003	0.1284 ± 0.045 *	0.0921 ± 0.028	0.1002 ± 0.001
Lachnospria	0.0471	0.0182 ± 0.001 *	0.0669 ± 0.005	0.0865 ± 0.004 *	0.0588 ± 0.001	0.0372 ± 0.001
Lachnoclostridiun	0.0153	0.0171 ± 0.0012	0.0117 ± 0.0003	0.0319 ± 0.0014 *	0.0067 ± 0.0006	0.0009 ± 0.0003 *
Bifidobacterium	0.0021	0.0008 ± 0.00007	0.0041 ± 0.00093	0.0017 ± 0.00032	0.0003 ± 0.00041	0.1061 ± 0.00602**
Lactobacillus	0.0265	0.0285 ± 0.0018	0.0332 ± 0.0004	0.0259 ± 0.0017	0.0313 ± 0.0048	0.0607 ± 0.0002 *

* $p<0.05$; ** $p<0.01$

Table S4. The list of gut microbiota which has the gene of SCFA produce.

ID	Name
CAG0007	Ruminococcus obeum CAG0007
CAG0010	Dorea longicatena CAG0010
CAG0012	Bacteroides caccae CAG0012
CAG0013	Parabacteroides merdae CAG0013
CAG0014	Bacteroidales bacterium CAG0014
CAG0015	Clostridium bolteae CAG0015
CAG0017	Clostridium sp. CAG0017
CAG0018	Bacteroides plebeius CAG0018
CAG0021	Bacteroides vulgatus CAG0021
CAG0022	Bacteroides stercoris CAG0022
CAG0023	Clostridiales bacterium CAG0023
CAG0026	Lachnospiraceae bacterium CAG0026
CAG0028	Flavonifractor plautii CAG0028
CAG0029	Lachnospiraceae bacterium CAG0029
CAG0030	Coprococcus comes CAG0030
CAG0031	Bacteroides uniformis CAG0031
CAG0032	Bacteroides thetaiotaomicron CAG0032
CAG0033	Ruminococcus sp. CAG0033
CAG0034	Oscillospiraceae bacterium CAG0034
CAG0035	Bacteroides fragilis CAG0035
CAG0036	Eubacterium eligens CAG0036
CAG0037	Eubacterium eligens CAG0037
CAG0039	Eubacterium hallii CAG0039
CAG0040	Megamonas funiformis CAG0040
CAG0041	Clostridiales bacterium CAG0041
CAG0045	Lachnospiraceae bacterium CAG0045
CAG0046	Bifidobacterium pseudocatenulatum CAG0046
CAG0048	Roseburia intestinalis CAG0048
CAG0050	Clostridiales bacterium CAG0050

CAG0051	Klebsiella pneumoniae subsp. pneumonia CAG0051
CAG0054	Clostridiales bacterium CAG0054
CAG0055	Subdoligranulum variabile CAG0055
CAG0056	Eubacterium eligens CAG0056
CAG0057	Lachnospiraceae bacterium CAG0057
CAG0058	Odoribacter splanchnicus CAG0058
CAG0062	Bacteroides cellulosilyticus CAG0063
CAG0063	Oscillospiraceae bacterium CAG0063
CAG0064	Bifidobacterium longum CAG0064
CAG0065	Bacteroides intestinalis CAG0065
CAG0066	Clostridiales bacterium CAG0066
CAG0067	Bacteroides vulgatus CAG0067
CAG0070	Blautia obeum CAG0070
CAG0075	Ruminococcus gnavus CAG0075
CAG0076	Bilophila wadsworthia CAG0076
CAG0077	Bacteroides coprocola CAG0077
CAG0079	Eubacterium rectale CAG0079
CAG0080	Clostridiales bacterium CAG0080
CAG0081	Ruminococcus torques CAG0081
CAG0082	Subdoligranulum variabile CAG0082
CAG0086	Bacteroides xylanisolvens CAG0086
CAG0089	Faecalibacterium prausnitzii CAG0089
CAG0090	Eubacterium eligens CAG0090
CAG0093	Eggerthella lenta CAG0093
CAG0096	Eubacterium eligens CAG0096
CAG0098	Klebsiella sp. CAG0098
CAG0100	Phascolarctobacterium succinatutens CAG0100
CAG0103	Ruminococcus sp. CAG0103
CAG0106	Faecalibacterium prausnitzii CAG0106
CAG0107	Subdoligranulum variabile CAG0107
CAG0109	Prevotella copri CAG0109
CAG0110	Faecalibacterium prausnitzii CAG0100

CAG0111	Clostridiales bacterium CAG0111
CAG0114	Ruminococcus albus CAG0114
CAG0115	Barnesiella intestinihominis CAG0115
CAG0116	Alistipes putredinis CAG0116
CAG0122	Adlercreutzia CAG0122
CAG0123	Lachnospiraceae bacterium CAG0123
CAG0124	Bacteroides vulgatus CAG0124
CAG0126	Ruminococcus bromii CAG0126
CAG0128	Odoribacter sp. CAG0128
CAG0130	Coprococcus sp. CAG0130
CAG0131	Clostridium sp. CAG0131
CAG0132	Lactobacillus salivarius CAG0132
CAG0133	Sutterella sp. CAG0133
CAG0134	Clostridiales bacterium CAG0134
CAG0138	Lachnospiraceae bacterium CAG0138
CAG0146	Sutterella wadsworthensis CAG0146
CAG0148	Bifidobacterium bifidum CAG0148
CAG0152	Clostridium bartlettii CAG0152
CAG0153	Eubacterium rectale CAG0153
CAG0155	Clostridium leptum CAG0155
CAG0158	Faecalibacterium sp. CAG0158
CAG0159	Ruminococcus lactaris CAG0159
CAG0165	Clostridiales bacterium CAG0165
CAG0166	Clostridiales bacterium CAG0166
CAG0169	Veillonella atypica CAG0169
CAG0171	Bifidobacterium adolescentis CAG0171
CAG0173	Roseburia hominis CAG0173
CAG0177	Ruminococcus champanellensis CAG0177
CAG0178	Bacteroides xylanisolvens CAG0178
CAG0179	Clostridiales bacterium CAG0179
CAG0180	Faecalibacterium prausnitzii CAG0180
CAG0182	Dialister sp. CAG0182

CAG0183	Faecalibacterium prausnitzii CAG0183
CAG0191	Megasphaera elsdenii CAG0191
CAG0201	Lactobacillus gasseri CAG0201
CAG0202	Oscillospiraceae bacterium CAG0202
CAG0203	Clostridiales bacterium CAG0203
CAG0207	Lactobacillus reuteri CAG207
CAG0218	Dialister succinatiphilus CAG0218
CAG0221	Oscillospiraceae bacterium CAG0221
CAG0224	Butyrate producing bacterium CAG0224
CAG0231	Veillonella parvula CAG0231
CAG0235	Clostridiales bacterium CAG0235
CAG0236	Lactobacillus delbrueckii CAG0236
CAG0246	Clostridiales bacterium CAG0246
CAG0248	Eubacterium ventriosum CAG0248
CAG0250	Ruminococcus bromii CAG0250
CAG0255	Oscillospiraceae bacterium CAG0255
CAG0256	Lactobacillus ruminis CAG0256
CAG0257	Oscillospiraceae bacterium CAG0257
CAG0260	Clostridium sp. CAG0260
CAG0264	Clostridium sp. CAG0264
CAG0272	Ruminococcus champanellensis CAG0272
CAG0274	Ruminococcus bromii CAG0274
CAG0281	Ruminococcaceae bacterium CAG0281
CAG0284	Burkholderiales bacterium CAG0284
CAG0287	Ruminococcus bromii CAG0287
CAG0289	Candidatus Arthromitus CAG0289
CAG0290	Ruminococcus torques CAG0290
CAG0292	Faecalibacterium prausnitzii CAG0292
CAG0294	Clostridiales bacterium CAG0294
CAG0300	Butyrate producing bacterium CAG0300
CAG0304	Ruminococcus bromii CAG0304
CAG0312	Oscillospiraceae bacterium CAG0312

CAG0313	Faecalibacterium cf. prausnitzii CAG0313
CAG0321	Oscillospiraceae bacterium CAG0321
CAG0331	Subdoligranulum variabile CAG0331
CAG0334	Lactobacillus crispatus CAG0334
CAG0339	Bifidobacterium breve CAG0339
CAG0341	Roseburia inulinivorans CAG0341
CAG0343	Prevotella stercorea CAG0343
CAG0353	Oscillospiraceae bacterium CAG0353
CAG0357	Clostridium sp. CAG0357
CAG0365	Bacteroides dorei CAG0365
CAG0366	Olsenella uli CAG0366
CAG0390	Clostridiales bacterium CAG0390
CAG0409	Lachnospiraceae bacterium CAG0409
CAG0421	Clostridiales bacterium CAG0421
CAG0439	Coprococcus catus CAG0439
CAG0450	Lachnospiraceae bacterium CAG0450

Table S5. Filtration on gut microbiota of different fermentation carbon source.

Fermentation carbon source	Class	Group	Parallel	Reference
EPDO-40	APS	P40	3	This study
EPDO-60	APS	P60	3	This study
EPDO-80	APS	P80	3	This study
Arabinoxylan	APS	WAX	3	To be published
Konjac glucomannan	APS	KGM	3	To be published
Dendrobium officinale oligo	OLIGO	DOO	5	To be published
Fructooligosaccharides	OLIGO	FOS	3	This study
Fructooligosaccharides	OLIGO	O	5	dio: 10.3390/nu14071476
Ganoderma lucidum oligosaccharides	OLIGO	H2	10	doi: 10.3390/nu14071476
Soluble starch	ST	SS	6	doi: 10.1021/acs.jafc.3c09327
Gelatinized Starch	ST	GS	3	doi: 10.1021/acs.jafc.3c09327
Lotus leaf flavonoids	YB	LLF	6	doi: 10.1021/acs.jafc.3c09327
Ferulic acid	PA	FA	3	doi: 10.1021/acs.jafc.3c09327

Table S6. The list of KEGG function analysis before and after filter of BLANK, P40, P60 and P80 group.

KEGG. Level 1	KEGG. Level 2	KEGG. Level 3	Group	LDA score
Before Filter				
Cellular Processes			BLANK	3.39573814
Human Diseases			BLANK	3.232148607
Environmental Information Processing	Signal transduction	Two component system	BLANK	3.221175779
Environmental Information Processing	Membrane transport		BLANK	3.177764934
Metabolism	Global and overview maps	Microbial metabolism in diverse environments	BLANK	3.050128392
Environmental Information Processing	Membrane transport	ABC transporters	BLANK	2.912304584
Cellular Processes	Cell motility	Flagellar assembly	BLANK	2.773652045
Human Diseases	Infectious disease bacterial		BLANK	2.732562398
Metabolism	Metabolism of cofactors and vitamins		P40	3.127202701
Metabolism	Nucleotide metabolism		P40	3.082712767
Environmental Information Processing	Membrane transport	Phosphotransferase system PTS	P40	2.821403987
Metabolism	Carbohydrate metabolism	Fructose and mannose metabolism	P40	2.703949132
Cellular Processes	Transport and catabolism	Lysosome	P40	2.550137204
Metabolism	Carbohydrate metabolism	Glyoxylate and dicarboxylate metabolism	P40	2.518499027
Metabolism			P60	4.435104122
Metabolism	Carbohydrate metabolism		P60	3.555469181
Metabolism	Glycan biosynthesis and metabolism		P60	3.218944432
Metabolism	Lipid metabolism		P60	2.977565631
Metabolism	Biosynthesis of other secondary		P60	2.968496359

	metabolites			
Metabolism	Carbohydrate metabolism	Galactose metabolism	P60	2.788846038
Metabolism	Glycan biosynthesis and metabolism	Other glycan degradation	P60	2.722985165
Cellular Processes	Transport and catabolism		P60	2.659602384
Organismal Systems			P60	2.645887216
Genetic Information Processing			P80	3.356333036
Genetic Information Processing	Replication and repair		P80	3.227494631
Metabolism	Energy metabolism		P80	3.04532519
Cellular Processes	Cell motility		P80	2.968592428
Metabolism	Carbohydrate metabolism	Starch and sucrose metabolism	P80	2.921755434
Genetic Information Processing	Translation	Ribosome	P80	2.7165657
Metabolism	Metabolism of cofactors and vitamins	Thiamine metabolism	P80	2.621237108
Metabolism	Energy metabolism	Photosynthesis	P80	2.595680778
After Filter				
Metabolism	Carbohydrate metabolism		BLANK	3.443323335
Metabolism	Global and overview maps	Microbial metabolism in diverse environments	BLANK	3.219368084
Metabolism	Glycan biosynthesis and metabolism		BLANK	3.187700052
Environmental Information Processing	Signal transduction	Two component system	BLANK	3.10719632
Metabolism	Amino acid metabolism		BLANK	3.080844802
Environmental Information Processing	Signal transduction		BLANK	3.064000125
Metabolism	Carbohydrate metabolism	Amino sugar and nucleotide sugar metabolism	BLANK	3.01917711
Human Diseases			BLANK	2.9699052

Genetic Information Processing			P40	3.281352533
Metabolism	Metabolism of cofactors and vitamins		P40	3.259971587
Metabolism	Global and overview maps	Biosynthesis of cofactors	P40	3.242690533
Genetic Information Processing	Translation		P40	3.127387267
Cellular Processes			P40	3.076020151
Metabolism	Nucleotide metabolism		P40	3.074494367
Genetic Information Processing	Translation	Ribosome	P40	3.044956296
Metabolism	Global and overview maps	Biosynthesis of amino acids	P60	3.411412542
Environmental Information Processing			P60	3.28958635
Environmental Information Processing	Membrane transport		P60	3.143148656
Environmental Information Processing	Membrane transport	ABC transporters	P60	3.085619137
Cellular Processes	Cellular community prokaryotes	Quorum sensing	P60	2.908059834
Metabolism	Carbohydrate metabolism	Starch and sucrose metabolism	P60	2.765415689
Cellular Processes	Cellular community prokaryotes		P60	2.718805436
Cellular Processes	Cell motility	Bacterial chemotaxis	P60	2.590339304
Metabolism	Carbohydrate metabolism	Galactose metabolism	P60	2.575123297
Metabolism	Carbohydrate metabolism	Pentose phosphate pathway	P60	2.559863466
Metabolism	Global and overview maps		P80	4.373897671
Genetic Information Processing	Replication and repair		P80	2.98870905
Genetic Information Processing	Translation	Aminoacyl tRNA biosynthesis	P80	2.636223686
Metabolism	Metabolism of cofactors and vitamins	Porphyrin and chlorophyll metabolism	P80	2.602359347
Metabolism	Energy metabolism	Methane metabolism	P80	2.523343963
